# EpicPred: predicting phenotypes driven by epitope-binding TCRs using attention-based multiple instance learning

**DOI:** 10.1093/bioinformatics/btaf080

**Published:** 2025-02-21

**Authors:** Jaemin Jeon, Suwan Yu, Sangam Lee, Sang Cheol Kim, Hye-Yeong Jo, Inuk Jung, Kwangsoo Kim

**Affiliations:** Interdisciplinary Program in Bioinformatics, Seoul National University, Seoul 08826, Republic of Korea; Interdisciplinary Program in Bioinformatics, Seoul National University, Seoul 08826, Republic of Korea; College of Computing, Yonsei University, Seoul 03722, Republic of Korea; Division of Healthcare and Artificial Intelligence, Korea National Institute of Health, Cheongju 28159, Republic of Korea; Division of Healthcare and Artificial Intelligence, Korea National Institute of Health, Cheongju 28159, Republic of Korea; School of Computer Science and Engineering, Kyungpook National University, Daegu 41566, Republic of Korea; Department of Transdisciplinary Medicine, Seoul National University Hospital, Seoul 03080, Republic of Korea; Department of Medicine, Seoul National University, Seoul 03080, Republic of Korea

## Abstract

**Motivation:**

Correctly identifying epitope-binding T-cell receptors (TCRs) is important to both understand their underlying biological mechanism in association to some phenotype and accordingly develop T-cell mediated immunotherapy treatments. Although the importance of the CDR3 region in TCRs for epitope recognition is well recognized, methods for profiling their interactions in association to a certain disease or phenotype remains less studied. We developed EpicPred to identify phenotype-specific TCR–epitope interactions. EpicPred first predicts and removes unlikely TCR–epitope interactions to reduce false positives using the Open-set Recognition (OSR). Subsequently, multiple instance learning was used to identify TCR–epitope interactions specific to a cancer type or severity levels of COVID-19 infected patients.

**Results:**

From six public TCR databases, 244 552 TCR sequences and 105 unique epitopes were used to predict epitope-binding TCRs and to filter out non-epitope-binding TCRs using the OSR method. The predicted interactions were used to further predict the phenotype groups in two cancer and four COVID-19 TCR-seq datasets of both bulk and single-cell resolution. EpicPred outperformed the competing methods in predicting the phenotypes, achieving an average AUROC of 0.80 ± 0.07.

**Availability and implementation:**

The EpicPred Software is available at https://github.com/jaeminjj/EpicPred.

## 1 Introduction

T-cell receptors (TCRs) are integral to the adaptive immune system, where they serve as the primary mechanism for T cells to recognize and bind to specific antigens. These antigens are presented by the major histocompatibility complex (MHC) in antigen-presenting cells (Carlberg and Velleuer 2022). The binding specificity of TCRs is determined by their variable regions that interact with epitopes and is crucial to the recognition of a wide array of pathogens. Understanding TCR–epitope interactions is vital, especially in the context of phenotypes (Lee *et al.* 2020). This understanding not only helps classifying immune responses but also plays a significant role in the development of vaccines and targeted immunotherapy.

**Figure 1.**
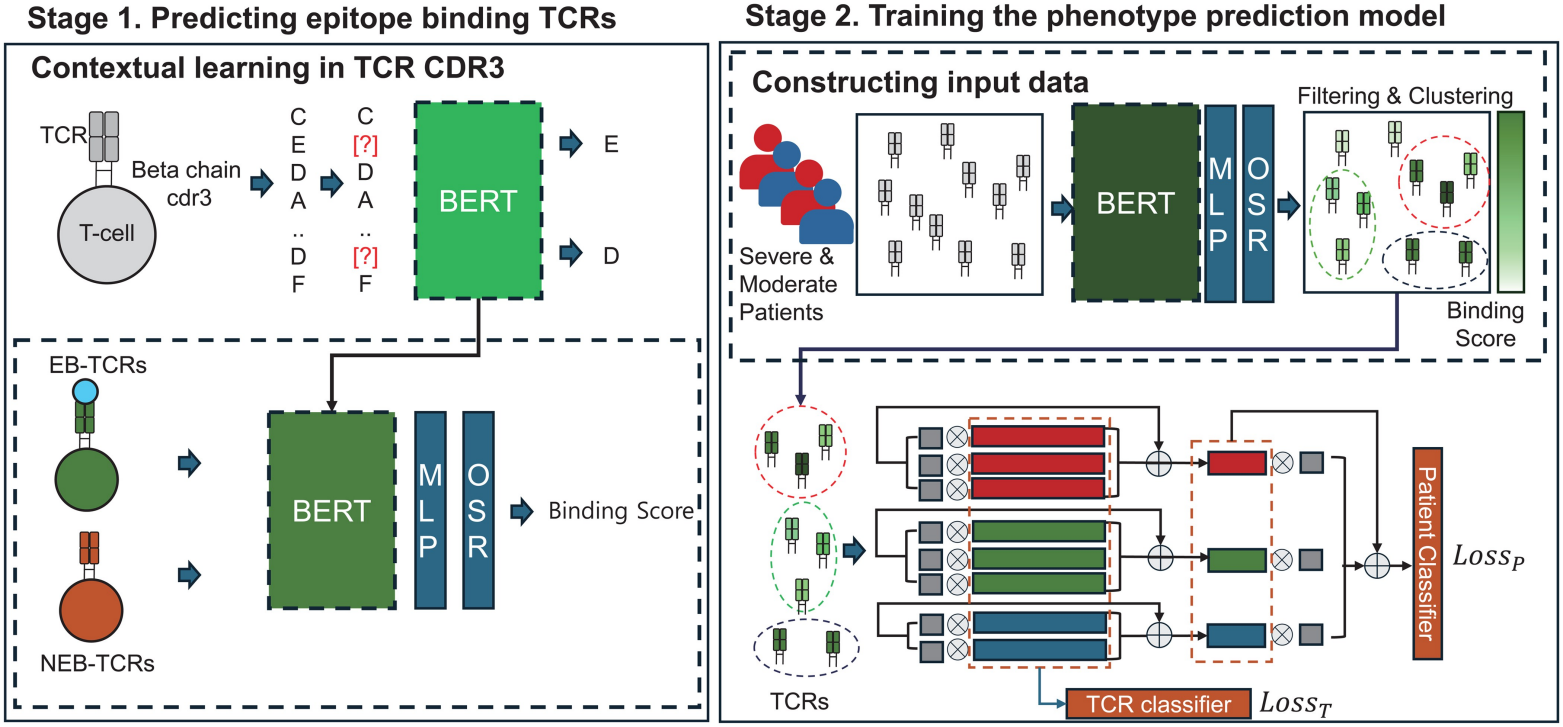
Workflow of the EpicPred model for sample phenotype prediction. EpicPred consists of two stages: (i) predicting epitope-binding TCRs and (ii) training the phenotype prediction model. In Stage 1, BERT is used to perform contextual learning on CDR3 sequences from TCRdb. The model integrates an MLP and an OSR layer, utilizing TCR–epitope data to predict 105 epitopes, enhancing the recognition of known and novel epitopes that are likely to bind to a TCR. In Stage 2, patient TCR CDR3 sequences are encoded using the BERT model trained in Stage 1. Binding scores are computed for each TCR, and sequences are filtered based on these scores before clustering. Epitope specific clustered TCRs are then used in an attention-based MIL model to predict the phenotype of a given input sample. Here, moderate and severe phenotype labels were shown as an example.

Recent research has extensively explored the way to predict epitopes with TCR sequences. Still, the task remains challenging due to the sheer diversity of epitopes, exceeding $10^{4}$ to $10^{6}$ distinct types, and the modest accuracy achieved in their prediction. The complexity of the immune system’s specificity is such that accurate epitope prediction requires sophisticated modeling. Recent developments have seen the advent of BERT-based approaches for TCR encoding, which have shown promise in this domain. Notable among these are models like TCR-BERT (Wu *et al.* 2021) and TCRconv (Jokinen *et al.* 2023), which leverage the BERT architecture to capture the intricate binding patterns between the TCR and its binding epitope. Beyond BERT-based approaches, ESM-2 (Evolutionary Scale Modeling) (Lin *et al.* 2023) and ProtT5 (Elnaggar *et al.* 2021) represent additional transformer-based methods that have demonstrated strong capabilities in understanding protein sequences. Recently, to accurately predict the binding affinity between epitope and TCRs, some of models are using TCRs that do not bind to epitopes, such as EPIC-TRACE (Croce *et al.* 2024) and MixTCRpred (Korpela *et al.* 2023). These models can be finetuned post-initial training to adapt to more detailed tasks offering versatile applications for epitope prediction that could potentially improve the accuracy and applicability of TCR analysis in immunological research. The utility of TCRs in phenotype prediction models has been predominantly focused on distinguishing cancerous conditions rather than assessing one’s phenotype. In COVID-19, models that exploit TCR data for predictive purposes are particularly scarce. Notable exceptions, such as DeepTCR (Sidhom *et al.* 2021) and MINN-SA (Kim *et al.* 2022), employ multiple instance learning (MIL) strategies. They often compress all TCR sequences into a single vector, potentially overlooking noncontributing TCRs and resulting in substantial information loss during dimension reduction. Here, we refer to noncontributing TCRs as the ones that do not bind to epitopes and thus are not considered as factors contributing to one’s severity progression. This highlights the need for a model that effectively identifies epitope-binding TCRs (EB-TCRs) to preserve relevant information. Models like DeepCAT (Beshnova *et al.* 2020) and DeepLION (Xu *et al.* 2022) address this by focusing on TCRs unique to patients compared to healthy individuals. However, these approaches often face challenges in generating unbiased training data and require substantial amounts of data to produce reliable results, which can be challenging. In this study, we present EpicPred, an attention-based deep learning framework designed to predict the severity of COVID-19 using the TCR beta chain sequences. Studies based on the TCR beta chain play a crucial role in identifying disease-specific features, such as predicting disease severity in COVID-19 patients (Xu *et al.* 2023) or developing bispecific antibodies targeting specific T-cell receptor beta variable regions for the treatment of T-cell cancers (Paul *et al.* 2021). EpicPred incorporates both epitope prediction and phenotype prediction, as illustrated in Fig. 1. Leveraging BERT-based learning for TCR sequences and employing open-set recognition (OSR) for the detection of uninformative TCRs, EpicPred was able to identify EB-TCRs in real datasets. Furthermore, using the attention-based MIL for phenotype prediction, our framework achieved better performance in classifying cancer samples and the severity of COVID-19 patients compared to competing methods. EpicPred provides a biological basis to identify which TCRs play a key role in influencing phenotype prediction, enabling us to understand the underlying immune mechanisms and the specific TCR–epitope interactions that contribute to particular phenotypic outcomes.

## 2 Datasets

The data used in this study were categorized for two distinct purposes. The first dataset was used for pretraining contextual learning and finetuning with epitope prediction. The second dataset includes TCR sequences from both single-cell and bulk data collected from cancer patients and COVID-19-infected patients. These datasets were used to evaluate the performance of phenotype prediction.

### 2.1 TCR beta chain CDR3 sequence dataset

We collected seven public datasets for pretraining (TCRdb, Chen *et al.* 2021), finetuning the epitope prediction models [IEDB (Vita *et al.* 2019), McPAS-TCR (Tickotsky *et al.* 2017), PIRD (Zhang *et al.* 2020), VDJdb (Shugay *et al.* 2018), 10× (Link to 10×), and neoTCR (Zhou *et al.* 2023)]. In our study, we utilized only the TCR beta chain CDR3 sequences for training, because the amount of beta chain sequences significantly exceeds that of the alpha chain. Moreover, the majority of related researches focus on the complementarity-determining region 3 (CDR3) of the TCR beta chain, as this region, being somatically generated, predominantly dictates the antigen-binding specificity. The details of each public dataset are summarized in Table 1. We applied filters for all the public datasets to exclude TCR sequences that were either shorter than 8 or longer than 22 length of amino acids. Additionally, sequences containing noisy CDR3 regions with nonstandard amino acids (i.e. those not among the canonical set of 20 amino acids) were removed. We included only the epitopes that had at least 200 known binding TCRs. The details of the TCR beta chain CDR3 sequences and binding epitopes used for training are provided in Supplementary Tables S1 and S2. For analyzing cancer samples, we incorporated neoTCR epitopes that had more than 10 TCRs per epitope due to their small number of annotated TCR–epitope instances (Supplementary Table S3). These TCRs are experimentally validated EB-TCRs. The 10× dataset predominantly consists of TCRs that are non-epitope-binding TCR (NEB-TCR).

**Table 1.** Datasets and number of TCRs and unique epitopes used for contextual learning and training the epitope-binding TCRs prediction model.

Dataset	Purpose	TCR sequences	Unique epitopes
TCRdb	Contextual learning	2 770 131	None
neoTCR	Epitope prediction	440	18
IEDB		125 048	93
McPAS-TCR		9504	23
PIRD		5463	15
VDJdb		4097	29
10X		100 000	None
Total		244 552	105

### 2.2 Phenotype sample dataset

For the analysis, four COVID-19 sample datasets were collected. They were sourced from the National Research Foundation of Korea [KNIH (Jo *et al.* 2022), *n* = 721], ISB-Swedish COVID-19 Biobanking Unit [SU (Su *et al.* 2020), *n* = 269], Adaptive Biotechnologies [Active (Athar *et al.* 2019), *n* = 308], and Cancer Research UK-Newcastle [CRUK (Stephenson *et al.* 2021), *n* = 49]. Additionally, we collected gastrointestinal cancer and lung cancer patient TCR-seq samples from Geneplus Technology Ltd in Shenzhen used in Lan *et al.* (2020). Detailed information of each dataset is shown in Table 2.

**Table 2.** Datasets and number of samples used for phenotype prediction.

Phenotype	Dataset	Data type	Healthy	Moderate	Severe
COVID-19	KNIH	Single-cell	81	283	44
		Bulk	96	364	92
	SU	Single-cell	16	192	43
	Active	Bulk	0	315	83
	CRUK	Single-cell	12	32	5

Phenotype	Dataset	Data type	Healthy	Cancer	

Cancer	Gastrointestinal cancer	Bulk	260	151	
	Lung cancer	Bulk	260	184	

## 3 Materials and methods

### 3.1 Workflow of EpicPred

EpicPred represents a novel approach to predict the phenotype of patients using EB-TCRs. EpicPred consists of two stages: (i) predicting EB-TCRs and (ii) training the phenotype prediction model.

#### 3.1.1 Stage1: Predicting EB-TCRs

To embed the TCR sequences into vectors, we used the BERT language model that employs a masked language model for learning language structures. Pretraining of the model was conducted using only TCR beta chain sequences collected from TCRdb. Each TCR sequence consists of an amino acid chain of length *L* drawn from an alphabet D of the 20 standard amino acids, i.e. D={A,C,S,…}. The model was configured with a hidden representation dimension size of 768, consistent with the standard BERT architecture. We adjusted the intermediate representation dimensionality to 1536, in contrast to the default BERT setting of 3072, to optimize computational efficiency and model capability. The model was designed with 12 attention heads and an equivalent number of transformer layers to ensure depth and complexity during learning. For pretraining, we used a batch size of 24 and a training duration of 100 000 stages, involving ∼2.4 million sequences, with early stopping implemented based on the validation loss. TCRs composed of *L* amino acids underwent transformation via BERT into an embedding matrix with dimensions of (L+2)×768. The matrix includes two additional features compared to the length of each TCR, represented by the classification [CLS] and separator [SEP] tokens. The [CLS] token, important for capturing the aggregate information of the sequence, plays a pivotal role in the classification finetuning process. Standard BERT training applies the cross-entropy loss function for each TCR, defined as *τ*, treating all tokens in the sequence equally when computing the error gradient. However, for TCR sequences, certain regions may hold more biological importance depending on their position within the sequence. To address this, our loss scaling function dynamically adjusts the model’s focus by increasing the importance of tokens located closer to the midpoint of a TCR sequence. This approach aligns with observations in previous studies (Newman and Riley 2007) that highlight the conserved and biologically informative nature of central regions within TCR sequences, which are critical for epitope recognition and binding. For each input TCR sequence *i* of length $L_{i}$, let $m_{i}$ denote the position of the masked token. We compute the weight *w_i_* for the TCR sequence *i* based on the token’s proximity to the midpoint of the sequence which is scaled by the scaling factor *δ* as follows, where *δ* is set to 0.6:

$$w_{i}=\text{log}\;\left(\frac{\frac{L_{i}}{2}}{\left|\frac{L_{i}}{2}-m_{i}\right|}+\epsilon \right)\times \delta .$$

Here, *ϵ* represents a small positive constant. Such weighted averaging ensures that sequences with more centrally located masked tokens contribute more to the overall loss, guiding the model to focus on these regions during training. The weight *w_i_* is used to compute the loss for Stage 1 (${\text{loss}}_{S1}$) as follows, where *N* is the number of TCR sequences:

$${\text{loss}}_{S1}=\frac{1}{N}\sum_{i=1}^{N}w_{i}\times \tau_{i}.$$

After contextual learning, a multilayer perceptron (MLP) combined with the maximal entropy loss (MEL) function (Vareto *et al.* 2024) were applied for epitope prediction, using the [CLS] token from each TCR sequence. The dataset used for this training process corresponds to the EB-TCR data described in Section 2.1. Specifically, TCR sequences binding to the 105 epitopes were selected and processed using a stratified 5-fold cross-validation method. Among the five folds, the model and data producing the best performance during testing were chosen for the final training and evaluation. A set of 105 epitopes, denoted as *G*, was taken, where each individual epitope is represented as *g*. The model was trained using the Adam Optimizer (learning rate = 0.001) to minimize the MEL. The MEL function calculates the binding probability using the $A_{Sm}$ [Adjusted Softmax with margin (Liu *et al.* 2016)] function, incorporating a margin (*m*) to increase the separation between different epitopes. The binding probability, *γ*, for a TCR–epitope pair is defined as:

$$\gamma =A_{Sm}({\text{logit}}_{g})=\frac{e^{{\text{logit}}_{g}-m}}{e^{{\text{logit}}_{g}-m}+\sum_{g\prime \neq g}e^{\mathrm{logi}t_{g\prime }}},$$

where ${\text{logit}}_{g}$ represents the raw score (logit) produced by the model for the epitope *g*, and the margin *m* adjusts the logits to improve classification. The margin reduces the distance of TCRs that bind to a common epitope and maximizes the distance of TCRs that bind to different epitopes, thereby enhancing the model’s ability to predict the correct binding epitope class. This function ensures that the logits are adjusted by a margin to amplify differences between the target epitope and all other epitopes (g′≠g). Following the computation of $A_{Sm}$, the MEL that utilizes the adjusted softmax function is defined as follows:

$$\text{MEL}=\{\begin{array}{cc} -\text{log}\;A_{Sm}(\text{LL}(R(t))) & \text{if}\;t\in G\\ -\frac{1}{\left|G\right|}\sum_{g=1}^{\left|G\right|}\;\text{log}\;A_{S}(\text{LL}(R(t))) & \text{if}\;t\notin G \end{array}.$$

Here, LL denotes the last layer of the MLP that generates logits, and R(t) represents the feature extraction values from a TCR sequence *t*. For TCRs from negative dataset, the loss function is designed to distribute the scores uniformly across all epitopes in the set *G*. This distribution helps the model to effectively distinguish TCRs that bind to previously unseen epitopes so that the classifier does not bias toward any specific known epitope making the classifier more general. Collectively, the output of Stage 1 is the probabilities for a TCR binding to each of the 105 epitopes and the encoded vector of each TCR output by the BERT model.

#### 3.1.2 Stage 2: Training the phenotype prediction model

Using the pretrained BERT model, specialized in contextual learning and finetuning with epitope prediction, each TCR beta chain CDR3 sequence (*T*) within a sample was encoded into a 768-dimensional vector for patient sample data. Based on the TCR–epitope-binding probabilities from Stage 1, we filtered out NEB-TCRs with low-binding probability. Subsequently, we applied K-means clustering to group similar TCR sequences. Clustered TCR sequences were used to train a phenotype prediction model. Here, two types of loss functions were used, which are based on cross-entropy. The first is the TCR-specific loss (Loss_*T*_) that focuses on determining whether a single TCR sequence within a sample is related to a phenotype, while the second is the sample-specific loss (Loss_*S*_), which focuses on detecting a group of TCRs related to a phenotype. We employed attention aggregation on the TCR sequences ($T_{1},T_{2},\ldots ,T_{N}$, where *N* represents the total number of TCR sequences) per patient, grouping them according to their clusters. This approach resulted in the creation of *K* distinct vectors ($C_{1},C_{2},\ldots ,C_{K}$, where *K* represents the total number of clusters), each representing a cluster representation vector. These *K* vectors were then subjected to another round of attention aggregation, which integrated them into a final sample representation vector *S*. We applied attention aggregation (Ilse *et al.* 2018) to each cluster with the following equation when *n* number of TCRs belonged to cluster *k*.

(1) $$w_{i}=\frac{\;\text{exp}\;\{Z^{⊤}\tanh(ZA_{i}^{⊤})\}}{\sum_{i=1}^{n}\;\text{exp}\;\{Z^{⊤}\tanh(VA_{i}^{⊤})\}}.$$

For the *i*th TCR attention weight, $A_{i}$ is calculated with the trainable parameters *Z* and *V*. Additionally, for each TCR sequence, the read count ratio, $r_{i}$, is included by dividing the individual TCR’s read count by the total read count of all TCRs for each sample. The output *C* from the attention layer is a weighted combination of the TCR sequence features in the *k*th cluster, computed by the following equation.

(2) $$C_{k}=\sum_{i=1}^{n}w_{i}T_{i}r_{i}.\;$$

With *K* cluster representation vectors, we applied attention aggregation to create the sample representation vector *S*. The loss in Stage 2 (${\text{loss}}_{S2}$) is created with sample-specific loss (${\text{loss}}_{S}$) and TCR-specific loss (${\text{loss}}_{T}$) with *S* and *T* as the following equation using cross-entropy loss.

(3) $${\text{loss}}_{S2}=(1-\lambda ){\text{loss}}_{S}+(\lambda ){\text{loss}}_{T}.$$

For each loss function, EpicPred used the weighted cross-entropy to account for class imbalances. Class weights in the loss function were adjusted dynamically, reflecting the class distribution within the dataset of interest.

### 3.2 Performance evaluation

The performance of EpicPred was compared with four prominent models in predicting phenotype: DeepTCR, DeepLION, DeepCAT, and MINN-SA, each offering unique insights into the phenotype state in terms of TCR sequences.

We aimed to evaluate how well each model predicted Healthy, Moderate, and Severe COVID-19 cases. Specifically, to obtain severity-related EB-TCRs, two types of binary classification were performed: (i) Healthy vs Severe and (ii) Moderate vs Severe. For the cancer dataset, the model was designed to classify samples into two classes: Healthy and Cancerous, distinguishing between normal and disease states. For bulk TCR data, we used the top 100 abundant TCRs based on their read counts. For DeepCAT, DeepLION, and MINN-SA, we address the challenge of training data imbalance by modifying the loss function to include weights proportional to group ratios, ensuring training on sample balanced datasets. DeepTCR provides an internal function to deal with unbalanced samples by applying class weights. In addition, we evaluated the impact of identifying NEB-TCRs and removing them from the training set in the pretraining Stage 1 by skipping such procedure. We employed 10-fold cross-validation, using 80% of the data for training, 10% for validation, and 10% for testing in each fold to ensure balanced model evaluation.

## 4 Results

### 4.1 Predicting EB-TCRs

**Figure 2.**
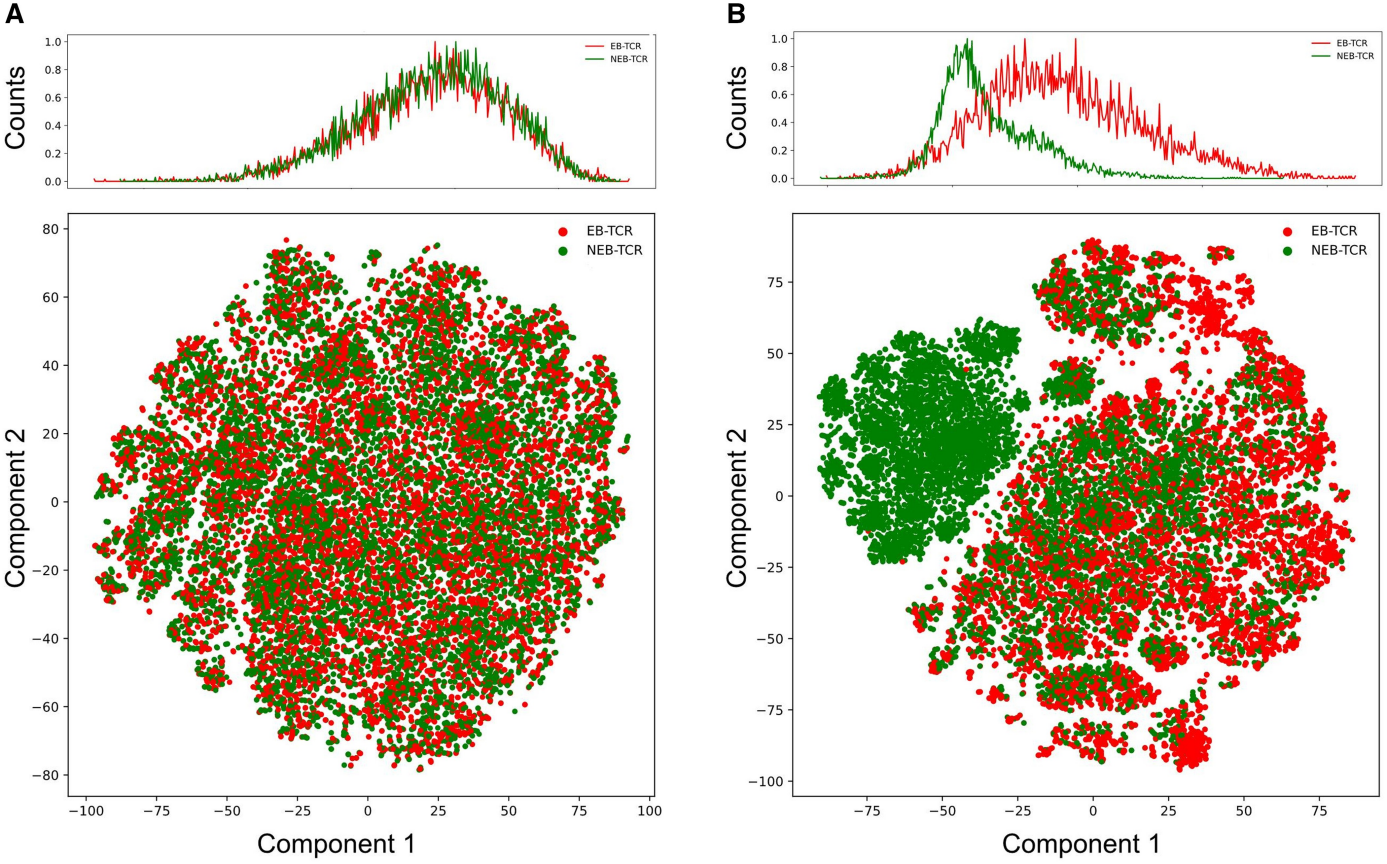
The frequency plot (top) and the t-SNE plot (bottom) visualize the embedded vectors of EB and NEB-TCRs. Each dot in the plot represents a TCR embedded sequence and top figure shows the frequency of the TCRs when collapsed on the t-SNE component 1. As shown, the EB and NEB-TCRs did not show difference (A) before the finetuning in Stage 1 but were clearly differentiated (B) after the finetuning.

In Stage 1, the goal was to accurately discriminate between EB-TCRs and NEB-TCRs, while also predicting the correct epitope class for each TCR. This approach enables the model not only to predict whether a TCR will bind to an epitope but also to score the likelihood of the interaction with high precision, facilitating the classification of TCRs based on their binding properties. The F1-score was evaluated on both a closed and an open set of TCR–epitope interactions using 5-fold cross-validation to compare the model’s performance across the two settings. The closed test set is comprised of only EB-TCRs, whereas the open test set includes both EB and NEB-TCRs. The task using the closed test set was to observe how many true TCR–epitope interactions were captured with different probability thresholds, as shown in Table 3. The variation in accuracy based on the sequence similarity between the training and test sets is depicted in Supplementary Fig. S1. The performance mildly dropped for dissimilar sequences but showed to be robust even for the most distant sequences, indicating that the model is general to some extent. Also, we compared the performance of predicting epitopes with existing models with TCR-BERT, DeepTCR, TCRconv, and TCRGP (Jokinen *et al.* 2021). We selected the top 10%, 20%, 30%, 50%, 70%, and 90% TCRs based on their binding probability scores and compared their F1-scores (Supplementary Table S4). The structural features of these models, including details about pretrained data and loss functions, are summarized in Supplementary Table S5. The models were trained using the same EB-TCR dataset, however, they do not consider NEB-TCR data, making EpicPred uniquely comprehensive in its evaluation of TCR specificity. Additionally, we evaluated the model’s performance for epitope prediction using different pretrained models to compare how the choice of pretrained model influences prediction accuracy (Supplementary Table S6). Also, it was tested with different percentages of the masking tokens (i.e. 10%, 15%, 20%, and 25%), which results are provided in Supplementary Table S7. For the open test set, the task was to measure the classification accuracy of discriminating the EB and NEB-TCRs, which results are shown in Table 4. In both results, by retaining a sufficient number of TCRs, the most robust accuracy was achieved at threshold 0.2 across 105 epitopes, with an F1-score above 0.7. We used this threshold for filtering TCRs with low probability for binding to an epitope before predicting phenotypes in single-cell TCR data. Additionally, the performance was compared under the same conditions with neoTCRs for the open test set, as presented in Supplementary Table S8. Since the sequences of EB-TCRs were further identified by the OSR added BERT model, their embedded vectors are expected to differ between the EB and NEB-TCRs. Indeed, the EB and NEB-TCRs embedded vectors showed clear difference before and after the training as shown in the t-distributed Stochastic Neighbor Embedding (t-SNE) plots in Fig. 2A and B, respectively. Each dot represents a TCR sequence embedded by the t-SNE components 1 and 2. The top figure represent the frequency of the cells when collapsed on the component 1.

**Table 3.** The F1-scores for predicting the epitopes that TCRs bind to.^a^

Closed test set						
Threshold (*γ*)	0.1	0.2	0.3	0.5	0.7	0.9
Precision	0.75 ± 0.01	0.80 ± 0.03	0.93 ± 0.09	0.96 ± 0.02	0.92 ± 0.03	0.98 ± 0.03
Recall	0.35 ± 0.01	0.65 ± 0.01	0.67 ± 0.02	0.82 ± 0.01	0.90 ± 0.03	0.95 ± 0.02
F1-score	0.40 ± 0.1	0.70 ± 0.05	0.75 ± 0.01	0.87 ± 0.02	0.92 ± 0.03	0.97 ± 0.01
Remain percentage	72%	40%	25%	17%	12%	10%

a A TCR was predicted to bind to an epitope if the predicted probability exceeded a threshold. The performance was tested for a range of different thresholds. The remaining percentage refers to the ratio of TCRs that have been predicted to bind to at least one of the 105 epitopes.

**Table 4.** Performance metrics for the open test set for classifying EB and NEB-TCRs, which were tested with different probability thresholds.

Open test set						
Threshold (*γ*)	0.1	0.2	0.3	0.5	0.7	0.9
Precision	0.78 ± 0.09	0.84 ± 0.04	0.88 ± 0.05	0.92 ± 0.02	0.94 ± 0.05	0.96 ± 0.01
Recall	0.78 ± 0.1	0.79 ± 0.09	0.65 ± 0.02	0.64 ± 0.01	red0.62 ± 0.08	0.61 ± 0.09
F1-score	0.80 ± 0.05	0.73 ± 0.05	0.72 ± 0.01	0.71 ± 0.05	0.71 ± 0.08	0.71 ± 0.01
Ratio of NEB-TCR	71%	25%	15%	10%	6%	4%

### 4.2 EB-TCR based phenotype prediction

**Figure 3.**
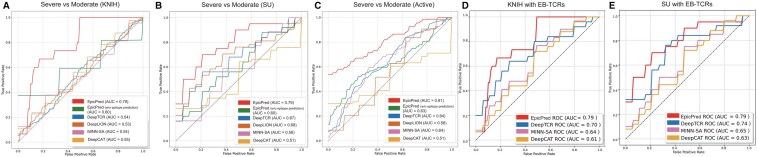
AUROC results predicting the moderate or severe health conditions of COVID-19 patients in the (A) KNIH, (B) SU, and (C) active datasets using EpicPred, EpicPred (without EB-TCR prediction), DeepLION, DeepTCR, DeepCAT, and MINN-SA. Panels (D) and (E) specifically compare the performance of models (DeepTCR, DeepCAT, and MINN-SA) using EB-TCRs on the KNIH and SU datasets.

Before comparing the model’s performance, we analyzed the CDR3 gene usage and diversity in the two COVID-19 datasets. Using the default pipeline (Park *et al.* 2023), which identifies differences in TCR repertoire, we found that the usage and diversity of CDR3 varied within the KNIH dataset (Supplementary Figs S2–S4). Notably, severe patients exhibited substantial differences in V and J gene usage and Chao diversity index (Chao *et al.* 2014) compared to healthy patients. While the previous study analyzed a different dataset (SU, Active dataset), we applied the same analysis specifically to the KNIH cohort. The phenotype prediction performance for patients with COVID-19 and cancer datasets was compared across the competing models that were trained with default parameters provided by each method. To address data imbalances, we adjusted the training process by employing weighted cross-entropy. Severity levels were predicted for the KNIH, SU, and Active datasets. Additionally, to assess the influence of EB-TCR prediction, we performed a comparative study by excluding the EB-TCR prediction component during training. TCR filtering was applied to exclude NEB like TCRs where the epitope prediction probability was below 0.2, which showed the most robust results in both the open and closed sets. To assess the impact of excluding NEB-like TCRs, we applied the subset of TCRs to other models, including DeepTCR, DeepCAT, and MINN-SA (Fig. 3 and Supplementary Table S9). DeepLION was not included in this comparison due to its requirement for a balanced number of TCRs per sample.

As shown in Fig. 3 and Table 5, there was a noticeable improvement in performance, particularly with single-cell data. Thus, we show that identifying EB-TCRs positively contributes to the clustering process, which allows for the simultaneous learning of interactions among similar TCRs. While the competing methods mainly focused on detecting cancerous samples, EpicPred was further applied to COVID-19 infectious patients to predict their severity. We retrained each model with the bulk cancer data for predicting healthy and cancer patients, where EpicPred yielded higher accuracy than the competing models as shown in Table 6. For each dataset, the embedding vectors of TCRs were clustered using K-means. The number of clusters *k* was set to 7 due to the overall high Area Under the Receiver Operating Characteristic (AUROC) in the three datasets as shown in Table 7.

**Table 5.** Performance of moderate and severe COVID-19 prediction with and without finetuning of EB-TCRs.

Dataset	KNIH (Single-cell)		KNIH (Bulk)		SU		Active	
Methods	EpicPred	EpicPred (w/o EB-TCR prediction)	EpicPred	EpicPred (w/o EB-TCR prediction)	EpicPred	EpicPred (w/o EB-TCR prediction)	EpicPred	EpicPred (w/o EB-TCR prediction)
F1-score (micro)	0.84 ± 0.03	0.81 ± 0.05	0.77 ± 0.14	0.51 ± 0.05	0.83 ± 0.06	0.68 ± 0.03	0.85 ± 0.05	0.81 ± 0.04
AUROC	0.79 ± 0.04	0.51 ± 0.14	0.72 ± 0.12	0.52 ± 0.01	0.79 ± 0.06	0.60 ± 0.09	0.81 ± 0.16	0.62 ± 0.08

**Table 6.** Performance comparison of six TCR based prediction models for classifying normal versus disease using cancer (Gastric, Lung cancer) and COVID-19 (KNIH) datasets.

Dataset	Metric	EpicPred	EpicPred (w/o EB-TCR prediction)	DeepTCR	DeepLION	DeepCAT	MINN-SA
Gastrointestinal cancer	F1-score (micro)	0.71 ± 0.05	0.62 ± 0.01	0.62 ± 0.08	0.57 ± 0.04	0.52 ± 0.02	0.62 ± 0.01
	AUROC	0.78 ± 0.04	0.72 ± 0.02	0.70 ± 0.05	0.54 ± 0.08	0.51 ± 0.05	0.58 ± 0.06
Lung cancer	F1-score (micro)	0.74 ± 0.11	0.72 ± 0.11	0.71 ± 0.04	0.67 ± 0.04	0.58 ± 0.05	0.6 ± 0.04
	AUROC	0.85 ± 0.03	0.82 ± 0.04	0.82 ± 0.01	0.71 ± 0.07	0.60 ± 0.03	0.62 ± 0.05
KNIH (scTCR)	F1-score (micro)	0.86 ± 0.01	0.75 ± 0.02	0.50 ± 0.10	0.58 ± 0.06	0.55 ± 0.01	0.65 ± 0.06
	AUROC	0.88 ± 0.01	0.78 ± 0.08	0.73 ± 0.09	0.52 ± 0.14	0.51 ± 0.11	0.50 ± 0.10
KNIH (Bulk)	F1-score (micro)	0.71 ± 0.05	0.58 ± 0.01	0.58 ± 0.13	0.60 ± 0.11	0.69 ± 0.06	0.69 ± 0.04
	AUROC	0.75 ± 0.01	0.71 ± 0.05	0.72 ± 0.09	0.56 ± 0.13	0.63 ± 0.12	0.68 ± 0.15

**Table 7.** Comparison of phenotype classification accuracy for each dataset using different number of clusters for *K*-means clustering.

K-means clustering AUROC							
Dataset	*k* = 3	*k* = 5	*k* = 7	*k* = 9	*k* = 11	*k* = 20	*k* = 30
Gastric cancer	0.75 ± 0.03	0.76 ± 0.04	0.78 ± 0.04	0.76 ± 0.01	0.76 ± 0.04	0.71 ± 0.08	0.70 ± 0.1
Lung cancer	0.88 ± 0.01	0.86 ± 0.08	0.85 ± 0.03	0.80 ± 0.05	0.80 ± 0.05	0.78 ± 0.05	0.80 ± 0.03
KNIH	0.83 ± 0.03	0.88 ± 0.01	0.88 ± 0.01	0.90 ± 0.05	0.88 ± 0.01	0.81 ± 0.06	0.82 ± 0.07

### 4.3 COVID-19 severity related cell sub populations with distinct EB-TCRs

**Figure 4.**
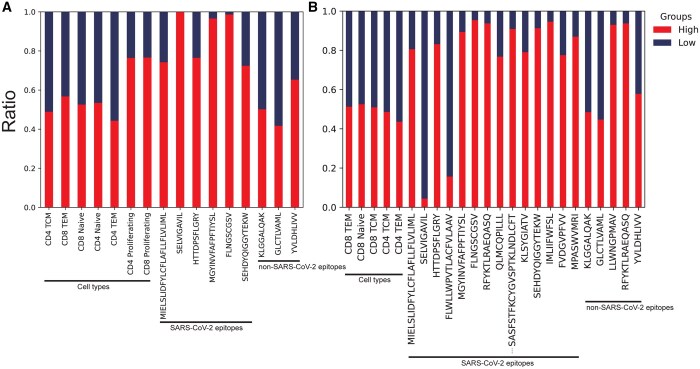
The ratio of cell types and predicted epitopes with high and low attention in the (A) KNIH and (B) SU datasets.

Utilizing single-cell RNA-seq samples and EpicPred’s attention scores, cells with high or low attention scores were searched. Cells with higher scores play an important role in predicting phenotypes, while those with lower scores have less impact on the prediction results. We categorized cells into two groups based on two percentiles: the top 30% representing cells with high attention and the bottom 30% representing low attention. Here, single-cell gene expression data of the KNIH and SU datasets were used that were comprised of 87 623 and 26 387 cells, respectively. Initially, genes expressed in fewer than three cells were removed resulting in 6253 and 7752 genes in the KNIH and SU datasets, respectively. We generated t-SNE plots using the TCR embedding vectors (1 × 768 dimension) and the single-cell embedding vectors (1 × 2500 dimension) (Supplementary Fig. S5). They were color coded by (A) cell type, (B) attention score, and (C) the predicted epitope labels. The cell types and epitope classes did not exhibit clear distinctions in the TCR t-SNE or the scRNA-seq t-SNE plots. However, there was a noticeable separation in attention score labels in both plots. To clarify the observation, Fig. 4 visualizes the proportions of cells that fall into the high and low attention groups. These proportions are categorized based on the cell types and the epitopes they bind to (referred to as cell features). Specifically, for each cell feature, the proportion of cells in the high attention group is calculated as follows: $\frac{\#\;\text{high}\;\text{attention}\;\text{cells}}{\#\;\text{high}+\text{low}\;\text{attention}\;\text{cells}}$. Only categories with a minimum of 1000 cells are shown. We divided cell features to three groups as “cell types,” “SARS-CoV-2 epitopes,” and “non SARS-CoV-2 epitopes,” and conducted *t*-tests between the high and low attention cells. It can be seen that only Severe Acute Respiratory Syndrome Coronavirus 2 (SARS-CoV-2) epitopes cell features significantly differed between the low and high attention groups as shown in Supplementary Fig. S6. For epitopes not associated with SARS-CoV-2, no significant difference was observed between the two attention groups. However, for SARS-CoV-2 epitopes, we observed a marked disparity in the ratios, indicating that cells recognizing SARS-CoV-2 epitopes tend to have higher attention scores. It implies that the attention mechanism of the EpicPred model may be particularly sensitive to epitopes related to SARS-CoV-2, suggesting potential for further investigation into immune responses to the virus. We conducted motif analysis on sequences with high attention scores. Using logomaker (Tareen and Kinney 2020), motif sequences were generated based on the TCR sequence length where similar motifs were captured across the KNIH, SU, and Active datasets. We find that the motifs suggest a commonality in the binding sites and sequence structures that contribute to high attention scores (Supplementary Fig. S7), and thus high TCR–epitope-binding possibility.

**Figure 5.**
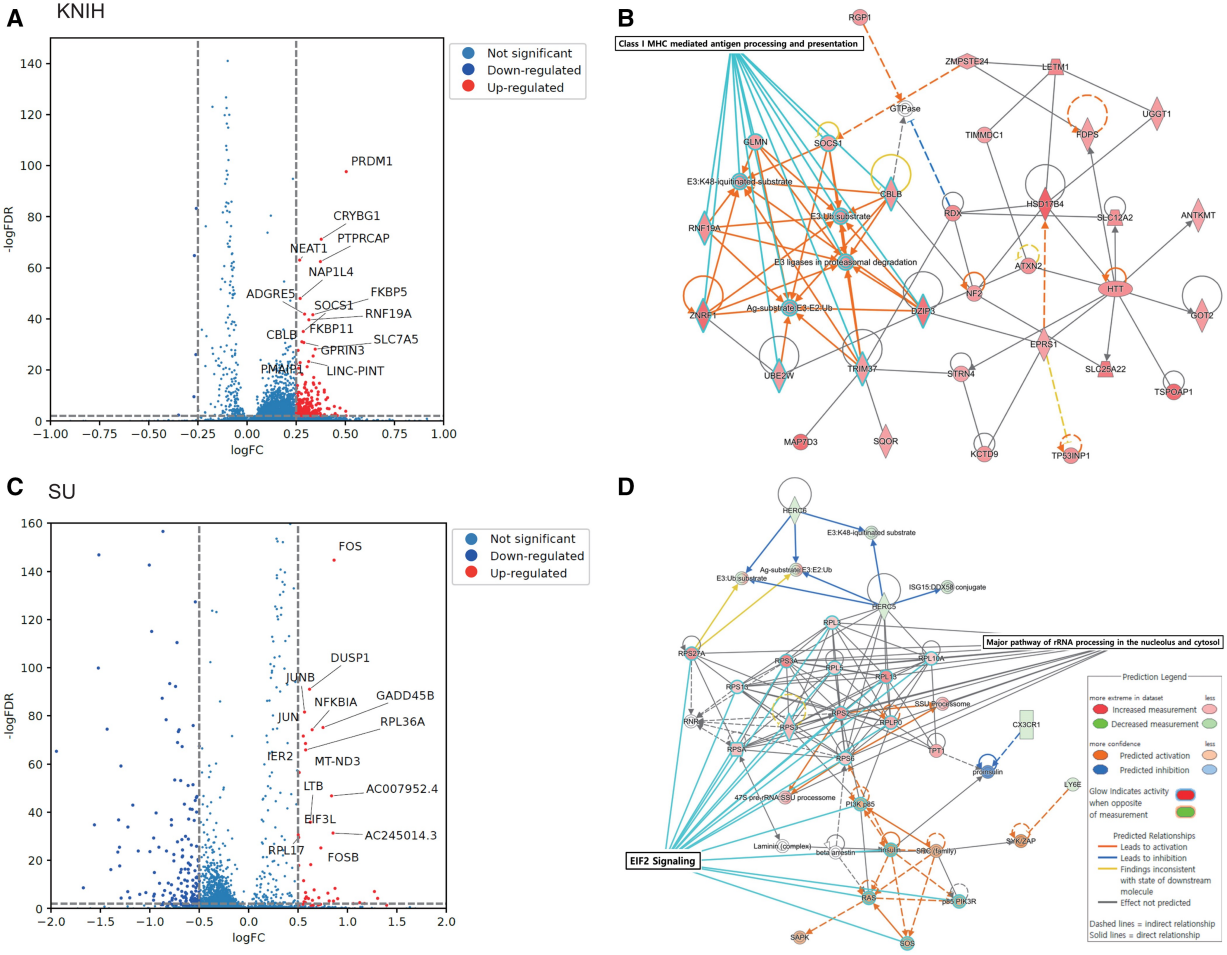
The Wilcoxon rank sum test results of the DEGs in cells from the high attention group in KNIH and SU datasets. Only genes with an adjusted *P*-value <.05 and logFC > 0.5 were considered. (A) and (C) show the DEG volcano plots. (B) and (D) the two largest networks identified from the DEG analysis of the SU and KNIH datasets using IPA. The color of each node indicates a change in expression: red represents upregulated genes, while green represents downregulated genes. The edges between nodes illustrate predicted relationships regarding biological function: orange signifies activation, blue denotes inhibition, and gray represents an effect that is not predicted.

We further investigated the biological characteristics of T cells with high attention scores by differentially expressed genes (DEGs) analysis. Wilcoxon rank-sum test was conducted between cells with high attention scores and all other cells. DEGs with an adjusted *P*-value below .05 and a log fold change >0.5 were searched, which are shown in Fig. 5A and C and list of genes are included in Supplementary Tables S10 and S11. A total of 564 and 126 DEGs were searched in the KNIH and SU datasets.

## 5 Discussion

EpicPred has been designed to overcome the constraints of traditional models that oversimplify TCR sequence data into a single vector representation, a process that can obscure the intricacies of individual TCRs and introduce biases in the analysis. By adopting epitope prediction tool before predicting phenotype, EpicPred adeptly aggregates TCRs based on their affinity with epitopes. Moreover, the integration of the OSR algorithm allowed us to improve the phenotype prediction, effectively compensating for the limitations in the scarcely available EB-TCR information. Furthermore, by examining the attention values, we were able to identify TCR sequences that particularly bind with SARS-CoV-2 epitopes, which were translatable to the single-cell transcriptome allowing us to identify the DEGs that were possibly affected by them. Especially, the disparity in epitope representation vectors and attention across the different severity levels showed that SARS-CoV-2 epitopes exhibit different TCR affinity.

In the single-cell datasets from KNIH and SU, DEGs were searched between the high attention and low attention cells. Downstream analysis on the DEGs was conducted using the QIAGEN Ingenuity Pathway Analysis (PA; QIAGEN Inc. link to IPA). Using IPA’s Core Analysis feature, significantly enriched canonical pathways were searched and constructed related gene networks as shown in Fig. 5C and D. Pathways with an absolute z-score of 2 or greater and an adj. *P*-value of .05 or less were considered statistically significant. The largest network identified in the KNIH dataset was the “Class I MHC-mediated antigen processing and presentation pathway,” which plays a crucial role in the immune response against viral infections, including SARS-CoV-2 (Zhang *et al.* 2021). The process of antigen processing and presentation is crucial for initiating and regulating the immune response against SARS-CoV-2, the virus responsible for COVID-19. It involves the presentation of viral antigens by MHC molecules to T cells, which is known to be essential for the adaptive immune response (Vabret *et al.* 2020). In the pathway analysis, neutrophils emerged as a key component of the innate immune response, which is also important to the pathophysiology of COVID-19. Elevated neutrophil counts and their activation are commonly associated with severe COVID-19 cases. Neutrophils migrate to infection sites, releasing neutrophil extracellular traps to contain pathogens (Barnes *et al.* 2020, Makatsariya *et al.* 2020). The pathway analysis results are listed in the Supplementary Tables S12 and S13. In the SU dataset, network analysis revealed the EIF2 signaling pathway as the largest network, and the EIF2AK4 (GCN2) response to amino acid deficiency pathway emerged as one of the significant canonical pathways in the analysis. These pathways are known to be essential for regulating cellular stress responses, particularly in the context of immune regulation and metabolic stress. Recent studies (Ravishankar *et al.* 2015, Policard *et al.* 2021), have shown that these pathways modulate immune responses, particularly in macrophages. It has been implicated in controlling inflammation and promoting tolerance to apoptotic cells, which is essential in preventing autoimmune responses. For instance, GCN2 is crucial for producing regulatory cytokines like IL-10 and TGF-*β*, which helps suppress inflammation and maintain immune tolerance.

To ensure the robustness of our model and assess potential overfitting, we validated the model on two independent datasets, SU and CRUK, after training on the KNIH dataset with 5-fold cross-validation. The model achieved an AUROC of 0.80 and an F1-score of 0.84 on the SU dataset, and an AUROC of 0.81 with an F1-score of 0.85 on the CRUK dataset showing that EpicPred is general.

Our investigation into EpicPred’s misclassification involved a detailed examination of samples with clinical severity that were incorrectly predicted as moderate, and vice versa. It involved an assessment of clinical features and WHO scores to elucidate the causes of such discrepancy. Supplementary Fig. S8A shows that severe cases misclassified as moderate presented clinical characteristics were inconsistent with the expected profile of severe COVID-19 patients. In contrast, Supplementary Fig. S8B indicates that samples erroneously predicted as severe exhibited clinical parameters typically associated with advanced Stages of the phenotype, particularly reflected in WHO scores. It implies that integrating clinical data with TCR profiling can improve the model’s diagnostic accuracy. For further research, we like to expand our focus beyond COVID-19, integrating BCR, TCR, and HLA type data with an extended epitope database for a more general interpretation of phenotype and epitope specificity.

All benchmarks were run on a machine equipped with 256 CPU cores under NUMA architecture, 512 GB of RAM, and an NVIDIA RTX A6000 GPU with 49 140 MiB of VRAM. On average, the pretraining phase took ∼7 h, the epitope prediction phase around 3 h, and the final sample prediction phase about 10 min.

## Supplementary Material

btaf080_Supplementary_Data

## Data Availability

TCR sequence data with Adaptive Biotechnologies immunoSEQ assays are available in the ImmuneCODE database at https://doi.org/10.21417/ADPT2020COVID. Single-cell TCR-seq data from ISB-Swedish COVID-19 Biobanking Unit25 are available at http://www.ebi.ac.uk/arrayexpress. Fifth Medical Center of PLA General Hospital data is available at http://ireceptor.irmacs.sfu.ca. The datasets of the COVID-19 cohort used in this are available online in the Clinical and Omics Data Archive (CODA) database. The Software EpicPred is freely available under the MIT license at https://github.com/jaeminjj/EpicPred.
