# Supplementary material for: EpicPred: predicting phenotypes driven by epitope-binding TCRs using attention-based multiple instance learning
Source: Bioinformatics. 2025 Feb 21;41(3):btaf080. doi: 10.1093/bioinformatics/btaf080 (PMC11879650; doi:10.1093/bioinformatics/btaf080)
Supplement: btaf080_Supplementary_Data [file btaf080_supplementary_data.zip › Supplementary_Figures.pdf]

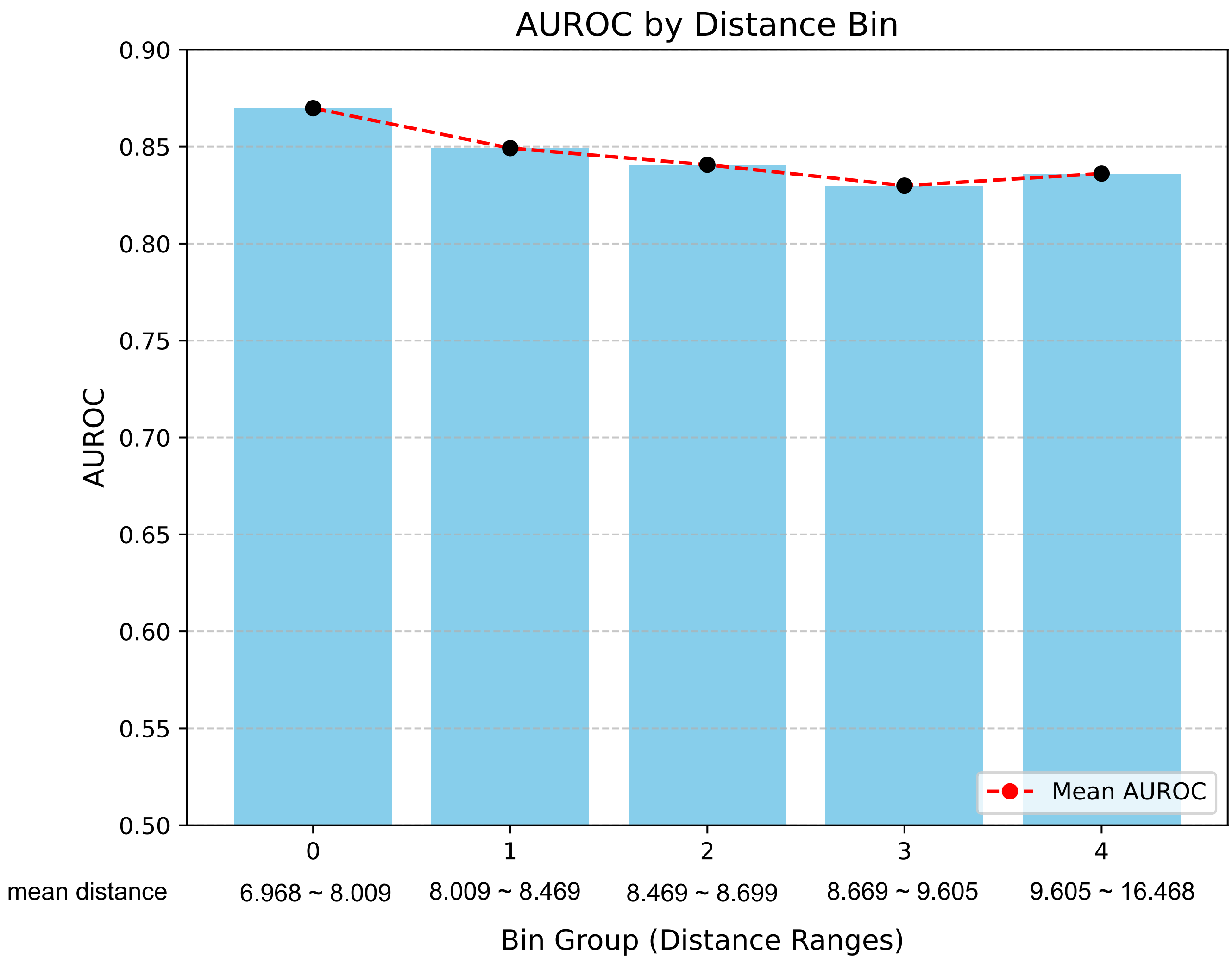

Supplementary Figure S1. Boxplot and line plot showing the AUROC performance of the model across five bins of test TCR sequences categorized by their levenshtein distances to the training TCR sequences. Bin 0 represents test sequences closest to the training sequences, while bin 4 represents test sequences farthest from the training sequences. The AUROC values demonstrate a decreasing trend as the distance between test and training sequences increases, indicating the model's reliance on sequence similarity for accurate predictions.

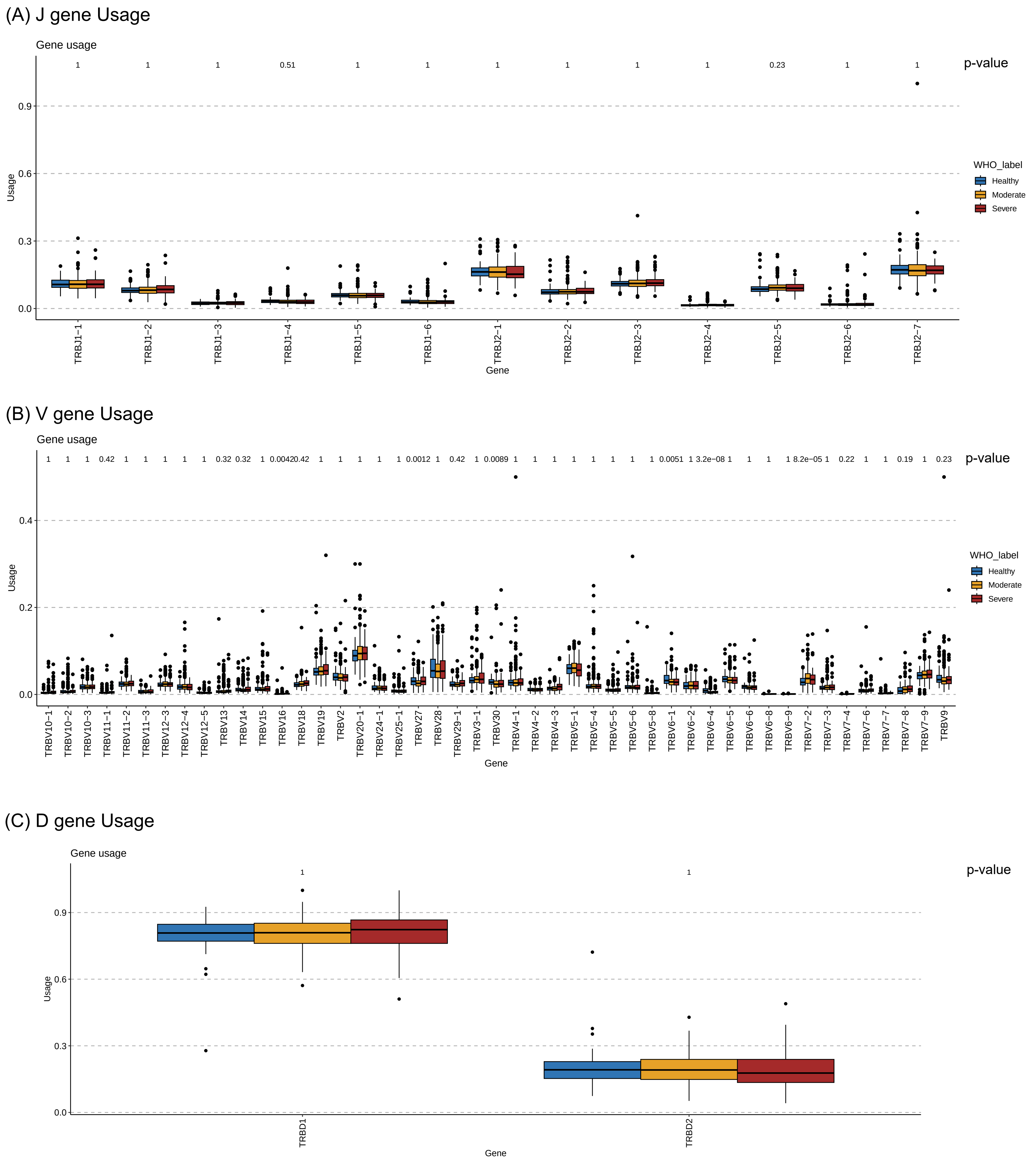

Supplementary Figure S2.Statistical Analysis of J gene (A), V gene (B), and D gene (C) Usage Variation Based on Severity Levels (WHO label).

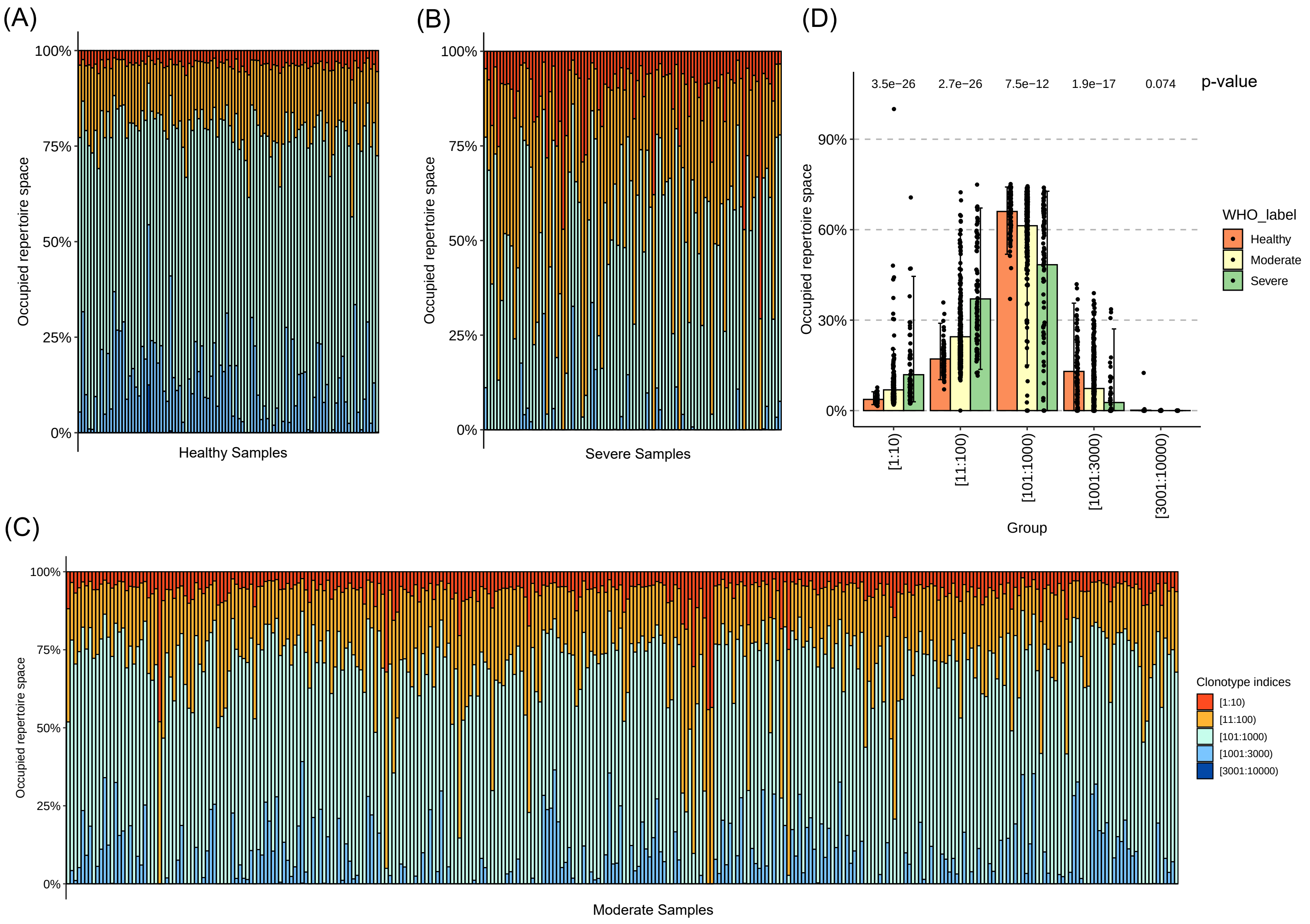

Supplementary Figure S3. (A), (B) and (C) depicting Repertoire Clonality for Severe, Moderate, and Healthy samples, calculated at various diversity levels (10, 100, 1000, 3000, 10000) using the 'top' method with Immunarch library in R package. (D) figure illustrates differences in clonality based on severity levels.

(A)

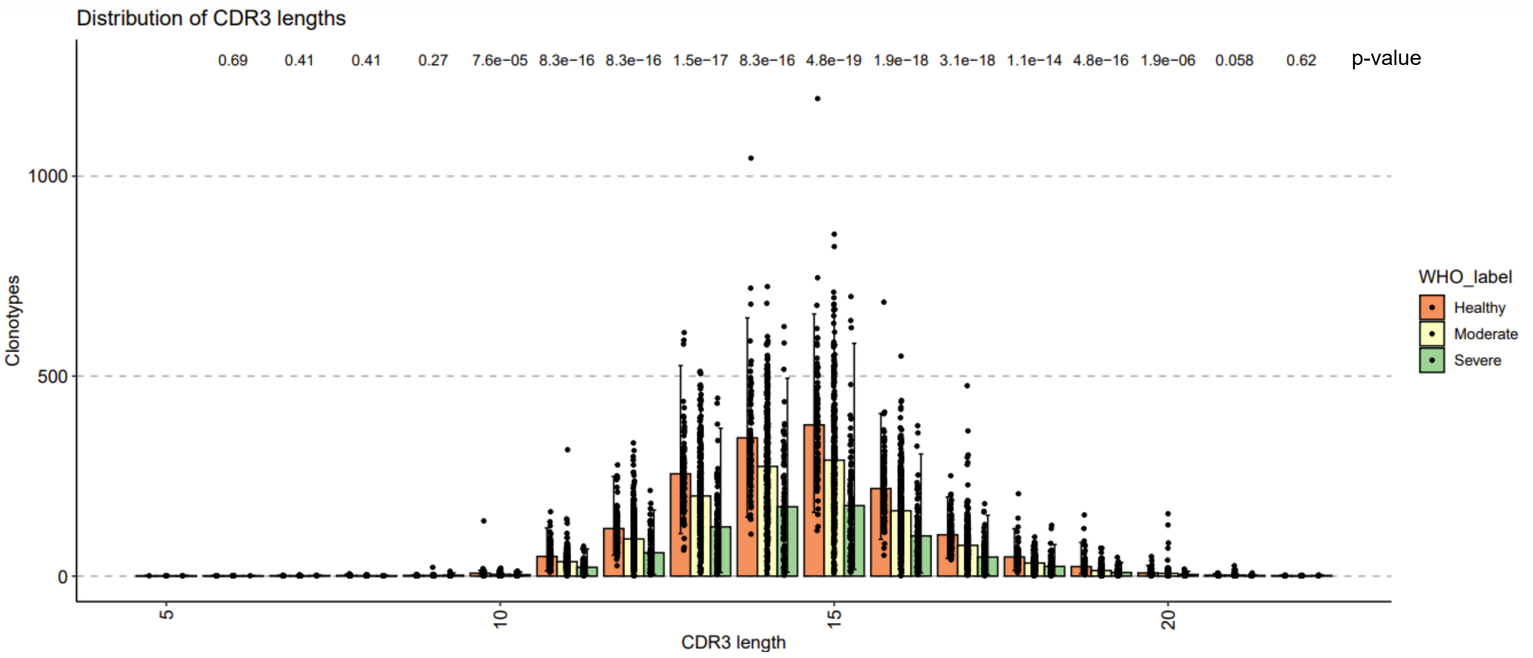

(B) Single Cell TCR

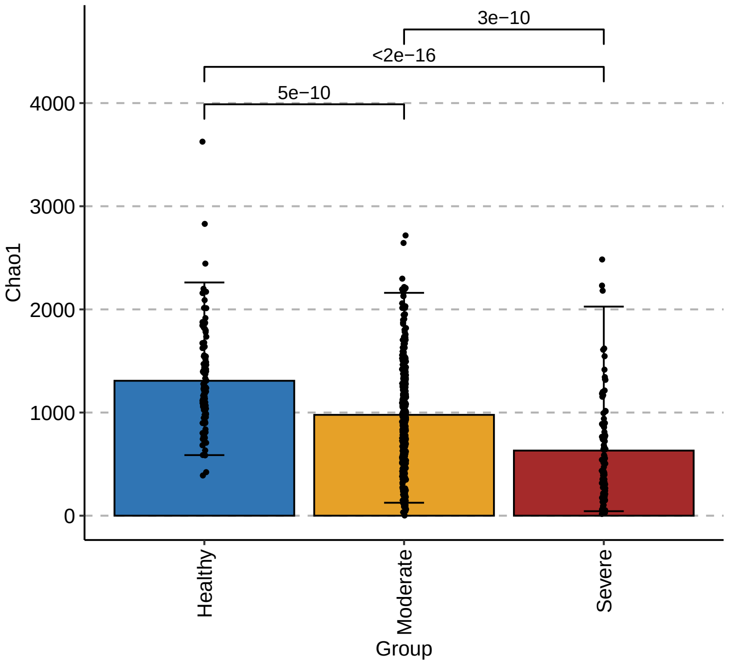

(C) Bulk TCR

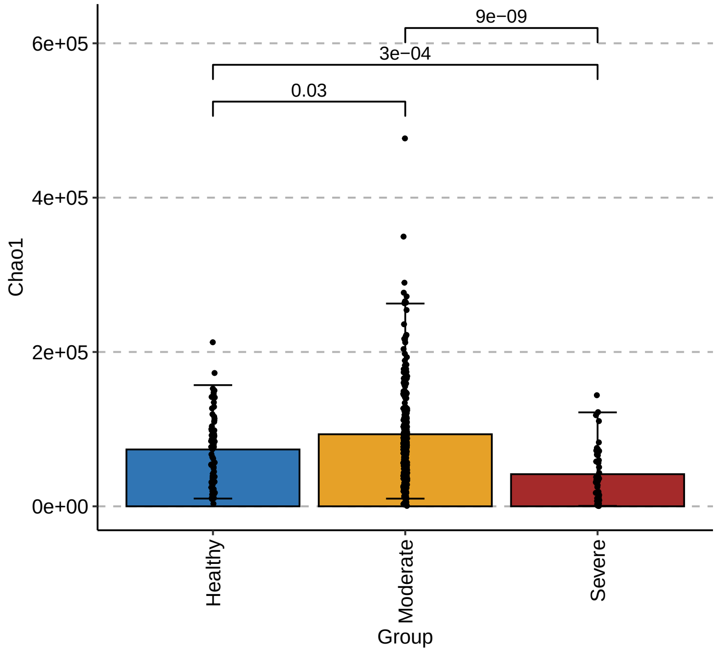

Supplementary Figure S4. (A) is distribution of CDR3 lengths across severity. Diversity differences in Single-cell TCR and Bulk TCR repertoires visualized using Chao1 values across varying severity levels.

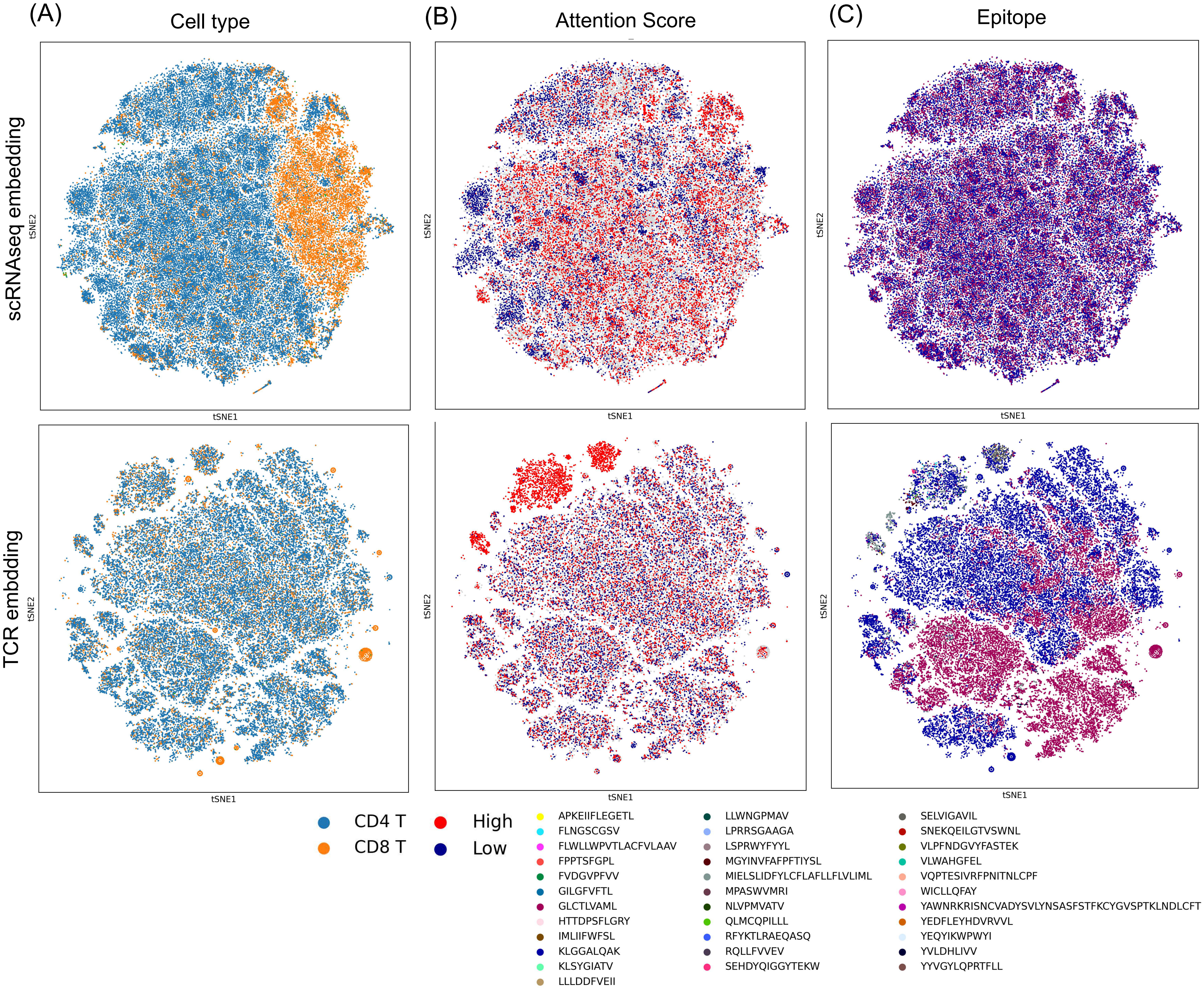

Supplementary Figure S5. Visualization of gene expression and TCR embeddings using t-SNE plots. (A) shows t-SNE plots of gene expression data, colored by CD4 and CD8 T cell labels. (B) depicts cells with high and low attention scores. (C) labels the cells by specific epitopes. The first row presents scRNA-seq expression data, while the second row shows t-SNE plots based on TCR embedding vectors.

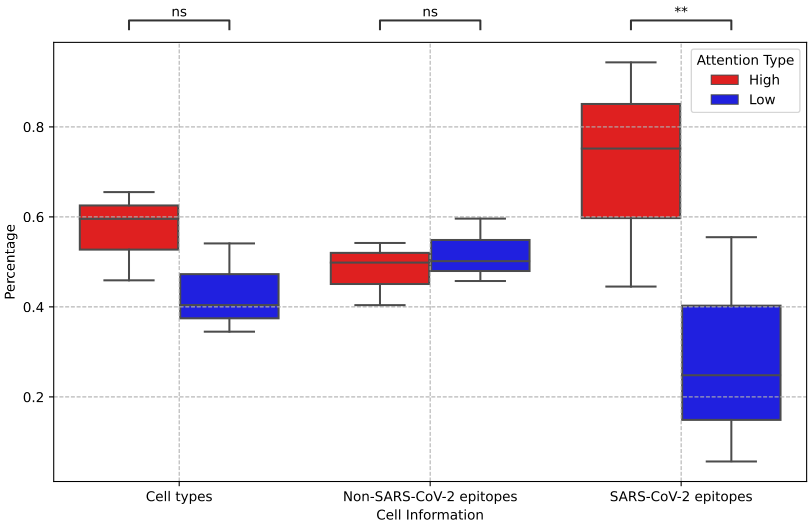

KNIH Severe vs Moderate

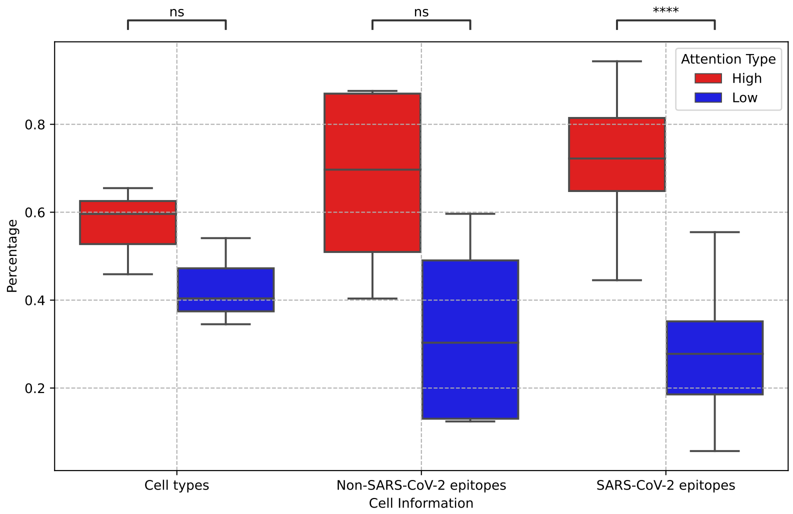

SU Severe vs Moderate

Supplementary Figure S6. Boxplot illustrating the distribution of cells with high versus low attention scores across different cell features, including cell types, non-SARS-CoV-2 epitope binding cells, and SARS-CoV-2 epitope binding cells. The proportion of cells in the high attention group is calculated for each cell feature, and the statistical significance of differences between high and low attention groups was assessed using a t-test. Adjusted p-values were calculated using the Benjamini-Hochberg method to control the false discovery rate (FDR). Significant results are denoted with * for adjusted p-values < 0.05, ** for adjusted p-values < 0.01, and *** for adjusted p-values < 0.001.

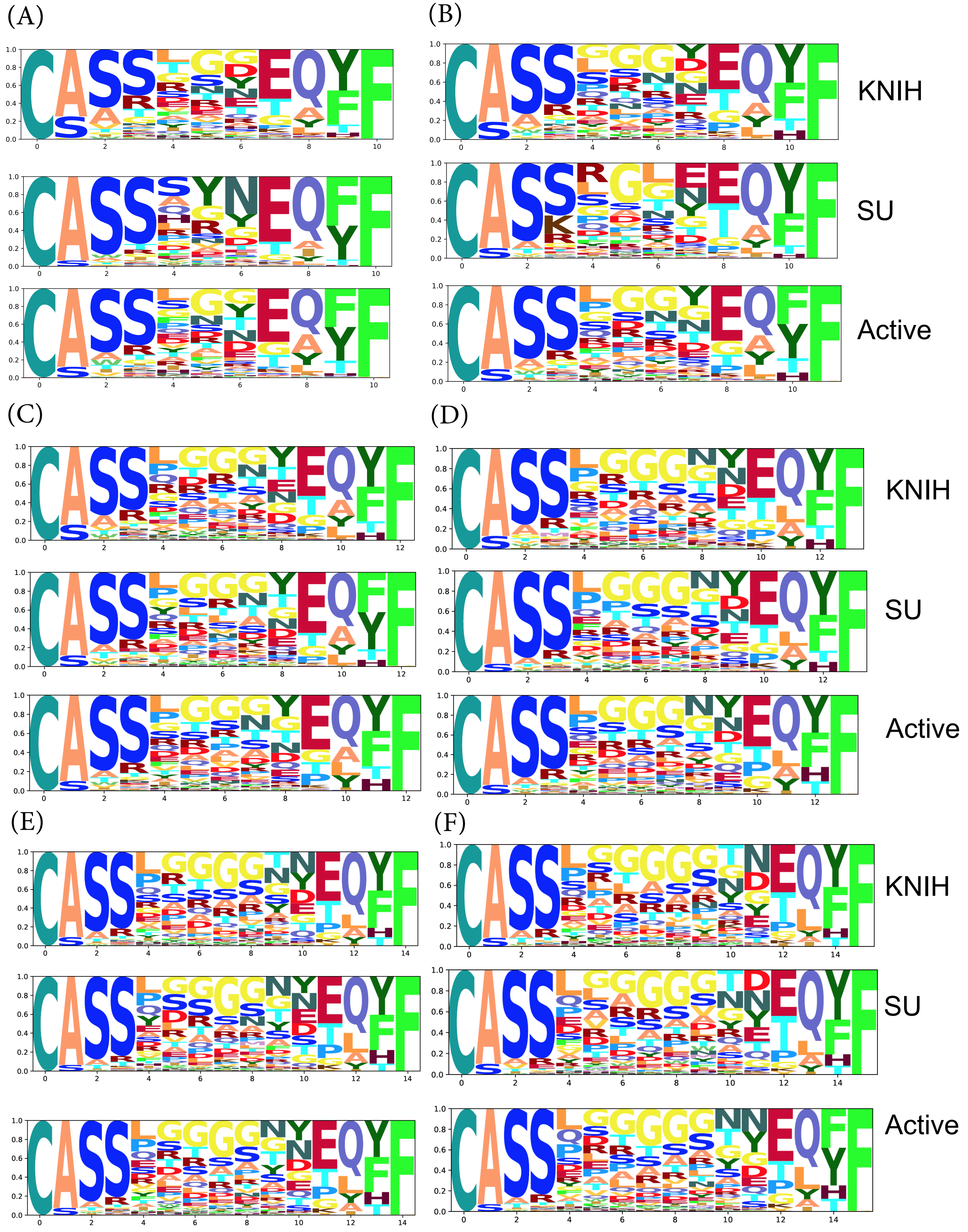

Supplementary Figure S7. Motif probability plot for the COVID-19 dataset from KNIH, SU, Active. Panels (A) to (F) represent sequences of lengths 11, 12, 13, 14, 15, and 16 respectively. The figure includes TCRs corresponding to the 30th percentile of attention scores.

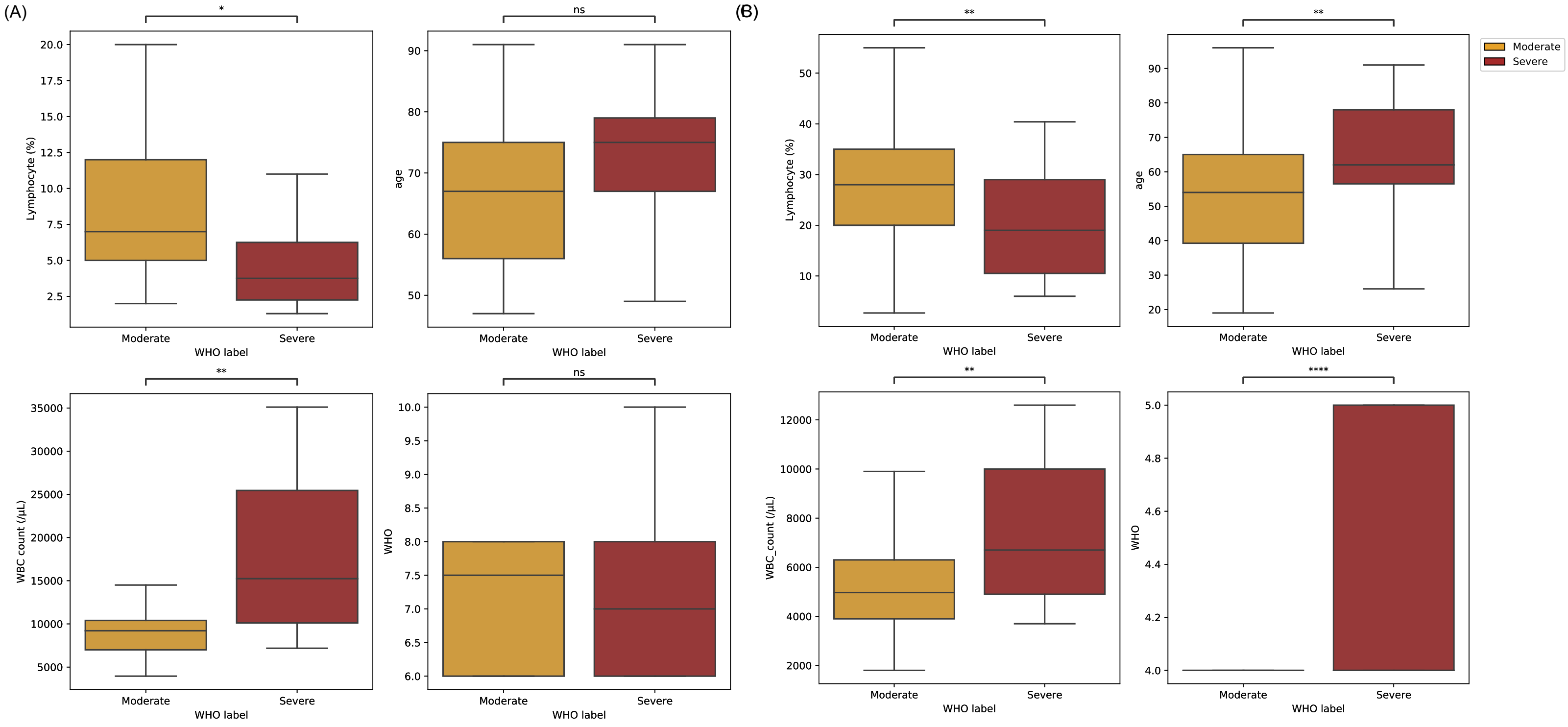

Supplementary Figure S8. Boxplot of difference clinical values (Lymphocyte, age, WBC count and WHO score) of misclassified as Severe groups (A) and correctly classified as Severe groups (B).
